# Neurodevelopmental Outcomes of Extremely Low Birth Weight Survivors in Johannesburg, South Africa

**DOI:** 10.3389/fped.2022.902263

**Published:** 2022-05-19

**Authors:** Tanusha D. Ramdin, Robin T. Saggers, Rossella M. Bandini, Yoliswa Magadla, Aripfani V. Mphaphuli, Daynia E. Ballot

**Affiliations:** ^1^Department of Paediatrics and Child Health, Charlotte Maxeke Johannesburg Academic Hospital and Faculty of Health Sciences, University of the Witwatersrand, Johannesburg, South Africa; ^2^PRINCE (Project to Improve Neonatal Care), School of Clinical Medicine, University of the Witwatersrand, Johannesburg, South Africa

**Keywords:** extreme low birth weight infants, neurodevelopmental, Sub-Saharan Africa, intensive care, Bayley Scales of Infant and Toddler Development-third edition

## Abstract

**Background:**

Improved survival in extremely low birth weight infants (ELBWI) in Sub-Saharan Africa has raised the question whether these survivors have an increased chance of adverse neurodevelopmental outcomes.

**Objectives:**

To describe neurodevelopmental outcomes of ELBWI in a neonatal unit in South Africa.

**Methods:**

This was a prospective follow-up study. All ELBWI who survived to discharge between 1 July 2013 and 31 December 2017 were invited to attend the clinic. Bayley Scales of Infant and Toddler Development (version III) were conducted at 9 to 12 months and 18 to 24 months.

**Results:**

There were 723 ELBWI admissions during the study period, 292 (40.4%) survived to hospital discharge and 85/292 (29.1%) attended the neonatal follow up clinic. The mean birth weight was 857.7 g (95% CI: 838.2–877.2) and the mean gestational age was 27.5 weeks (95% CI 27.1–27.9). None of the infants had any major complication of prematurity. A total of 76/85 (89.4%) of the infants had a Bayley-III assessment at a mean corrected age of 17.21 months (95% CI: 16.2–18.3). The mean composite scores for cognition were 98.4 (95% CI 95.1–101.7), language 89.9 (95% CI 87.3–92.5) and motor 97.6 (95% CI 94.5–100.6). All mean scores fell within the normal range, The study found 28 (36.8%) infants to be “*at risk*” for neurodevelopmental delay.

**Conclusion:**

Our study demonstrates good neurodevelopmental outcome in a small group of surviving ELBWI, but these results must be interpreted in the context of the high mortality in this group of infants.

## Background

In South Africa, preterm birth occurs in ~15% of all births–that is, one in seven infants are born premature (1, 2). Extremely low birth weight infants (ELBWI) are defined as having a birth-weight <1,000 g. Fortunately, advances in neonatal care over the last few decades have improved the survival of premature infants, raising concerns about their morbidity and neurodevelopmental outcomes (3).

The World Health Organization (WHO) reported that three quarters of premature infants can be saved with feasible and cost-effective care, such as antenatal steroids, kangaroo mother care and breastfeeding (1, 2). Yet, there is a notable difference in the rates of survival and neurodevelopmental impairment in ELBWI in high income countries (HICs) compared to low and middle income countries (LMICs). In HICs, the mortality rate of extremely premature infants is <10%, compared to 70–90% in LMICs (3, 6). These differences are attributed to several factors; namely population demographics, antenatal and neonatal care, post-hospital discharge guidelines and resource availability (4).

Decreasing mortality rates in ELBWI in LMICs by establishing a high level neonatal intensive care unit (NICU) might incur a huge financial burden as well as risk of inequity and diverting resources from more mature neonates. However, a study conducted by Ballot et al. (5) at a tertiary hospital in Johannesburg, showed an improved survival rate of 50% between 2006/2007 and 2013 by giving surfactant and nasal continuous positive airway pressure (NCPAP) to infants between 750 and 900 g. Similarly, a study in Cape Town, reported an increased survival of 75% in infants with a birthweight 500–1,000 g who received NCPAP and surfactant therapy (6). This highlights that simple, low-cost interventions provided to ELBWI can significantly improve survival rates.

Survivors may experience a range of short term morbidities including bronchopulmonary dysplasia (BPD), retinopathy of prematurity (ROP), intraventricular haemorrhage (IVH) and long term neurodevelopmental morbidities, which may include developmental delay, cerebral palsy, blindness, and deafness (7–9). A literature review by Jarjour et al. (7) concluded that nearly half of surviving ELBWI will have significant neurodevelopmental disability on short and long term follow up.

In LMICs there is very limited data on long term neurodevelopmental outcomes of ELBWI (10). Ballot et al. (11) conducted a study in a similar setting in 2013 assessing neurodevelopmental outcomes in very low birth weight infants (VLBWI) using the Bayley Scales of Infant and Toddler Development III (Bayley-III), in which a subset of ELBWI had no evidence of developmental delay. This was in agreement with reports of good developmental outcomes of ELBWI in India, a low income country (12). A Cochrane review by Spittle et al. (13) of 25 randomized trials indicated that early neurodevelopmental interventions, like parent-infant interactions and physiotherapy interventions based on principles of neuro-developmental therapy, has benefits for preterm infants in combined cognitive and motor outcomes.

This study aimed to determine the neurodevelopmental outcome of ELBWI in a middle income country in Sub-Saharan Africa.

## Methods

This was a prospective follow-up study of ELBWI born between 1 July 2013 and 31 December 2017. The study was conducted at the neonatal unit of a tertiary hospital in Johannesburg, South Africa. Charlotte Maxeke Johannesburg Academic Hospital (CMJAH) is a public sector hospital that serves a low socioeconomic community that does not have access to private health insurance All ELBWI who survived to hospital discharge were invited to enrol. Enrolment was done at the first clinic visit.

The ELBWI study group were seen at the study clinic every 3 months until the corrected age of 24 months. To improve rates of follow-up, text messages were sent to parents of enrolled participants as reminders of follow-up appointments. Transport costs were refunded and defaulting patients were traced and rebooked where possible.

Appropriately trained paediatricians and physiotherapist performed the developmental assessments using the Bayley Scales of Infant and Toddler Development, version III (Bayley-III) (11). The Bayley III was validated in the same setting (14). The first Bayley-III assessment was conducted between 9 and 12 months; the second between 18 and 24 months (if patient still attended the follow up clinic). The Bayley-III assessment would be done at the next visit if a child defaulted a study clinic visit. The Bayley-III scores were calculated using the age corrected for prematurity. The gestational age was assessed by maternal menstrual history and clinical assessment using the Ballard score (11). The Cronbach's alpha interclass correlation between different observers for neurodevelopment assessment was 0.89 (14). Infants with congenital abnormalities that were likely to affect neurodevelopment, for example Trisomy 21, were subsequently excluded from the study.

Developmental delay was classified “*at risk”* if a composite Bayley-III score was below 85 on any of the cognitive, language or motor sub-scales and as “*delayed”* if a composite Bayley-III score was below 70 on any of the sub-scales (14). Cerebral palsy was diagnosed if there was a delay in motor milestones together with abnormal movement and/or posture (14). Hearing and vision were indirectly assessed as part of the Bayley-III language and motor assessment. Where developmental problems were identified, the child was referred for appropriate intervention by the allied medicine team (physiotherapy, occupational therapy and speech therapy).

### Data Management

Data were entered and managed using Research Electronic Data Capture (REDCap™) software, hosted by the University of Witwatersrand (15). Maternal variables included demographics, antenatal care (ANC), obstetric history, place and mode of delivery. Neonatal variables included gestational age, birth weight, sex, duration of ventilation and stay, neonatal morbidity, late sepsis and outcome.

### Statistical Analysis

The data were exported into SPSS version 23 (IBM, USA) for statistical analysis. The latest Bayley-III score for each child was used for analysis. The composite cognitive, language and motor scores were used as outcome variables. If continuous variables were normally distributed, the data was described using mean and 95% confidence intervals (95% CI). Skewed data was described using median and interquartile range (IQR). Categorical variables were described using frequency and percentages. Survivors and non-survivors were compared–continuous variables were compared using unpaired *t* test or Mann Whitney U depending on the data distribution. Categorical variables were compared using Chi Square. Only valid cases were analysed for each variable (i.e., missing data was excluded).

### Ethical Considerations

Written informed consent was obtained from the parents of each participant prior to study enrolment. The Human Research Ethics Committee of the University of the Witwatersrand, Johannesburg, approved the study (reference numbers M120623 & M170702).

## Results

**Figure 1:**
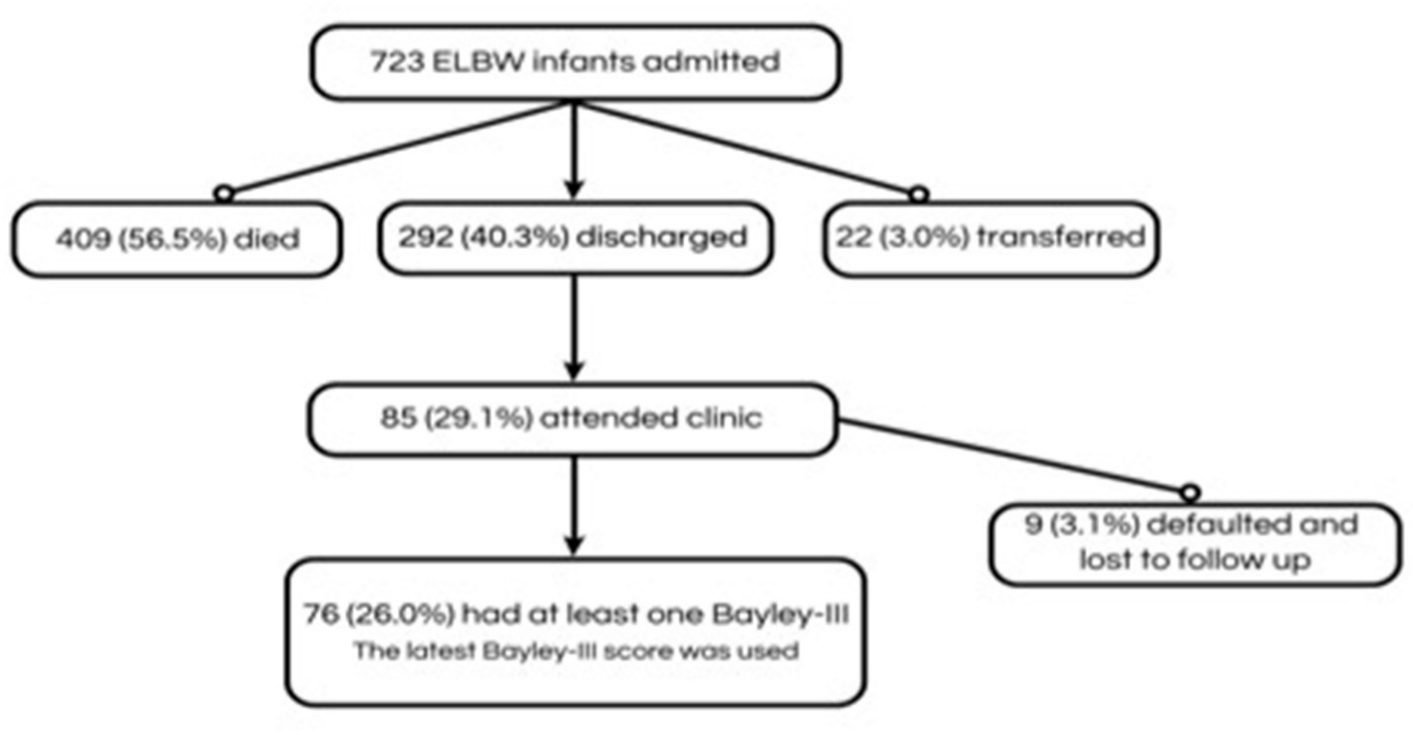
Participants included in the study of neurodevelopmental outcomes of extremely low birth weight infants at a tertiary hospital in Johannesburg, South Africa, 1 July 2013 and 31 December 2017.

There were 723 ELBWI admitted to the unit during the study period. The characteristics of survivors and non-survivors is shown in Table 1. The overall cohort of ELBWI had a mean birth weight of 822.28 grams and gestational age of 27.09 weeks. The majority of ELBWI received NCPAP (74.6%). ELBWI who died were of lower birth weight and gestational age and more likely to have complications of prematurity (Table 1). Patient selection is shown in Figure 1. Of 292 infants who were discharged, 85 (29.1%) attended the follow-up clinic.

**Table 1:** Clinical characteristics and risk factors associated with mortality in ELBWI at a tertiary hospital in Johannesburg, South Africa.

	**Total *N* = 723**	**Died *N* = 409**	**Survived *N* = 314**	***P*-value**
Birthweight (grams)	822.28 ± 103.7 Median and IQR	771.11 ± 125.2 Median and IQR	873.45 ± 82.2 Median and IQR	***P*** **<** **0.001**
Gestational age (Weeks)	27.09 ± 2.01 Mean and SD	26.48 ± 2.17 Mean and SD	27.7 ± 1.86 Mean and SD	***P*** **=** **0.014**
Late onset sepsis	260 (36.0%)	101/409 (24.7%)	159/314 (50.6%)	***P*** **<** **0.001**
Intraventricular haemorrhage grade 3 or 4	44 (6.1%)	31/409 (7.6%)	13/314 (4.1%)	*P* = 0.06
Patent ductus arteriosus	89 (12.3%)	34/409 (8.3%)	55/314 (17.5%)	***P*** **<** **0.001**
Necrotising enterocolitis	55 (7.6%)	30/409 (7.3%)	25 / 314 (8.0%)	*P* = 0.778
Gender = male	313 (43.3%)	183/409 (44.7%)	130/314 (41.4%)	*P* = 0.405
5 min Apgar 5 or less	133 (18.4%)	96/364 (26.3%)	37/288 (12.8%)	***P*** **<** **0.001**
Nasal continuous positive airways pressure	531 (74.6%)	281/409 (68.7)	258/314 (82.2%)	***P*** **<** **0.001**

*IQR, Interquartile Range; SD, Standard deviation. Bold values indicate the significant variables*.

Neurodevelopmental assessment was conducted in 76 of the 85 (89,4%) infants who attended the clinic. The characteristics of these infants are shown in Table 2.

**Table 2:** Clinical and demographic characteristics of 76 extremely low birth weight infants assessed with Bayley Scales of Infant and Toddler Development (Version III) at a tertiary hospital in Johannesburg, South Africa.

**Variable**	**Frequency**	**Percentage**
**Maternal characteristics**
Antenatal care (at least one visit)	68	89·5
Antenatal steroids (any number of doses)	46	60·5
Antenatal magnesium sulphate for preeclampsia	10	13·2
Maternal hypertension	33	43·4
Maternal HIV	20	26·3
Chorioamnionitis	1	1·3
**Mode of delivery**
Delivered by elective or emergency caesarean	55	72·3
section
**Neonatal characteristics**
Resuscitation at birth	38	50·0
Five minute Apgar score below 6	10	13·1
Respiratory distress syndrome	74	97·4
Surfactant therapy	62	81·6
Nasal CPAP without mechanical ventilation	48	63·2
Mechanical ventilation	13	17·1
Early onset sepsis (<72 h of life)	2	2·6
**Complications of prematurity**
Patent ductus arteriosus	9	11·8
Necrotising enterocolitis stage 2 or 3	4	5·3
Anaemia requiring blood transfusion	56	73·7
Late onset sepsis (>72 h of life)	36	47·4
Intraventricular haemorrhage Grade 1 or 2	14	18·4
Steroids for chronic lung disease	27	35·5
Retinopathy of prematurity stage 3 or more	1	1·3
**Outcomes**
Kangaroo mother care	48	63.2
Exclusively breastfed on discharge	32	42.1

The Bayley-III assessment was conducted at a mean corrected age of 17.21 months (95% CI: 16.2–18.3). The mean composite scores are shown in Table 3. All mean scores fell within the normal range, but the composite language score was the lowest. We did not diagnose cerebral palsy in any of the infants. These findings were similar to the neurodevelopmental outcomes in a group of low risk term infants from the same unit (11) (see Table 3).

**Table 3:** Composite score of Bayley Scales of Infant and Toddler Development (Version III) in 76 extremely low birth weight and low-risk term infants at a tertiary hospital in Johannesburg, South Africa.

	**Extremely low birth weight infants**		**Low risk term infants**	
**Domain**	**Mean composite score**	**95% Confidence intervals**	**Mean composite score**	**95% Confidence intervals**
Cognitive	98·4	95·1–101·7	92.2	89.4–95.0
Language	89·9	87·3–92·5	94.8	92.5–97.1
Motor	97·6	94·5–100·6	98.8	96.8–101.0

We found 28 of 76 ELBWI infants (36.8%) infants to be “*at risk*”−17 in one domain; seven in two domains and four in three domains. The language domain had the most “*at risk*” infants, with 22 of 76 infants at risk. Of the 28 “*at risk”* infants, we classified two (2.6%) as disabled–one in all three domains and one in two domains (motor and language). There were no cases of cerebral palsy identified.

## Discussion

Our study provides information on neurodevelopmental outcomes of ELBWI in a middle- income country (MIC) in Sub Saharan Africa. The mean cognitive, language and motor Bayley-III scores were all within the normal range and there were no cases of cerebral palsy. The neurodevelopmental outcome of the ELBWI was similar to that in a group of low-risk term infants from the same setting (11). These findings are encouraging and provide evidence that some ELBWI in Sub Saharan Africa can have a normal outcome. This type of information is useful to inform neonatal protocols and parental counseling in this context.

Importantly, more than one third of the ELBWI were identified as “*at risk”* and two of them were disabled. There were no disabled infants and 25.6% with cognitive delay, 16.2% language delay and 5.2% motor delay in the group of low-risk term infants from the same setting (11). The identification of at risk and disabled infants enabled the early referral to rehabilitation services. Appropriate early neurodevelopmental intervention may assist in decreasing the burden of a lifetime of functional disability after preterm birth (13). Ideally ELBWI require longer follow up to the age of 8 years or more to discover more unfolding sequalae.

### Cautious Interpretation

These promising results must be interpreted with caution. There is strong selection bias in this study due to the high mortality in this group of high-risk infants. The normal developmental outcome in our study is in strong contrast to report rates of handicap in ELBWI in HICs (16). However, none of the surviving ELBWI in our study had any serious complication of prematurity and would be expected to have a normal outcome. Previous research in our unit has shown a strong association between complications of prematurity and mortality (17). The smallest and sickest babies do not survive to discharge. Our survival rate of ELBWI 40% is less than half that reported in HICs but similar to other MICs (3, 6). Therefore, our study evaluated a group of good survivors who may be expected to have normal development.

We also had a low rate of follow up in our study. Only one third of the ELBWI survivors attended the follow up clinic. We did not evaluate reasons for failing to attend the follow up clinic, but these may include financial issues, lack of transport, relocation to other provinces or countries and parents unable to miss work to attend the clinic. Unfortunately, we do not know the developmental outcome of the infants that were lost to follow-up.

### Study Limitations

Imprecise gestational age determination was a limitation of the study. The gestational age was based on a Ballard assessment in most cases and not accurate dates, nor first trimester antenatal sonar. Access to first trimester antenatal sonar is very limited in our setting. Most mothers present to antenatal clinic book during second or third trimester of pregnancy. Second and third trimester accuracy in determining gestational age varies from 10 to 30 days, respectively. Mothers usually present late in labour to the hospital. Therefore, in our setting birth weight is routinely used instead of gestational age (11, 18).

The high loss to follow up of neonatal survivors is a challenge in our setting. We did not evaluate the reasons for loss to follow up but CMJAH is a tertiary hospital in the inner city of Johannesburg. The low rate of follow up could be related to the migrant population. More than one third of the CMJAH mothers come from neighboring provinces or countries to give birth at CMJAH and return homes after their babies are discharged. Many mothers return to work, and it is extremely difficult for them to take off from work to the attend clinic. Due to limited financial resources, there is no dedicated social worker or nurse to ensure that babies return to follow up clinic.

The ELBWI survivors in our study were treated in a specialised tertiary centre and are therefore not generalizable to lower levels of care. Most ELBWI are born and treated in regional and district hospitals without the same level of neonatal care.

So too there are limitations on the assessment tools used: the Bayley-III may underestimate impairment among extremely preterm infants (19). Studies have found that the mean score of cognitive and motor development was close to normal (means 96.9–100.4), which is higher than expected when compared to the prior data of similar cohorts tested with Bayley-II (11). Furthermore, the infants' first or home language are not English therefore the composite language scores may have been lower than expected and relatively lower than other composite scores. However, the language score was low but within normal limits. Low language score was also found in similar studies done at our settings (11). Cranial ultrasound of the study group showed grade 1 and 2 IVH and is unlikely to be associated with low language score.

## Conclusion

Although these results are encouraging, there is strong selection bias in our study sample. The survival rate of ELBWI was low, the surviving infants did not have major complications of prematurity and most survivors did not attend neonatal follow up clinic. Our results show that this small sub-group of ELBWI had normal developmental outcomes at approximately 24 months but does not reflect neurodevelopmental outcome in all ELBWI in sub-Saharan Africa.

The study also demonstrated some of the significant challenges relating to this type of follow up in LMICs. Our results highlight the need for a properly resourced prospective cohort follow up study of ELBWI in LMICs, to have reliable neurodevelopmental outcome data in this group of vulnerable infants. Understanding these outcomes of prematurity will provide important data to neonatologists, treating doctors, families and policy makers and will guide decision making regarding the provision of intensive care for ELBWI.

## Data Availability Statement

The raw data supporting the conclusions of this article will be made available by the authors, without undue reservation.

## Ethics Statement

The studies involving human participants were reviewed and approved by Human Research Ethics Committee of the University of the Witwatersrand, Johannesburg, (reference numbers M120623 and M170702). Written informed consent to participate in this study was provided by the participants' legal guardian/next of kin.

## Author Contributions

TR performed data collection and wrote up the various drafts for publication. DB conceptualized the study, performed data collection and analysis, assisted in the write up, and review of the various drafts for publication. RS, YM, AM, and RB assisted with data collection. RS and RB assisted in the review of the final draft for publication. All authors have read and approved the final version of this manuscript.

## Funding

A Self-Initiated Research Grant from the South African Medical Council funded this study (DB). The grant assisted with travel costs for parents and participants to follow up clinic visits.

## Conflict of Interest

The authors declare that the research was conducted in the absence of any commercial or financial relationships that could be construed as a potential conflict of interest.

## Publisher's Note

All claims expressed in this article are solely those of the authors and do not necessarily represent those of their affiliated organizations, or those of the publisher, the editors and the reviewers. Any product that may be evaluated in this article, or claim that may be made by its manufacturer, is not guaranteed or endorsed by the publisher.
